# Supplementary material for: Splice-Junction-Based Mapping of Alternative Isoforms in the Human Proteome
Source: Cell Rep. Author manuscript; Available in PMC 2020 Jan 15. (PMC6961840; doi:10.1016/j.celrep.2019.11.026)
Supplement: 2 [file NIHMS1546469-supplement-2.zip › DF1/PXD010154/Lung/Lung_48_CAPN12_NSMPLSSRPYIQLVDEEAGVGAGR.pdf]

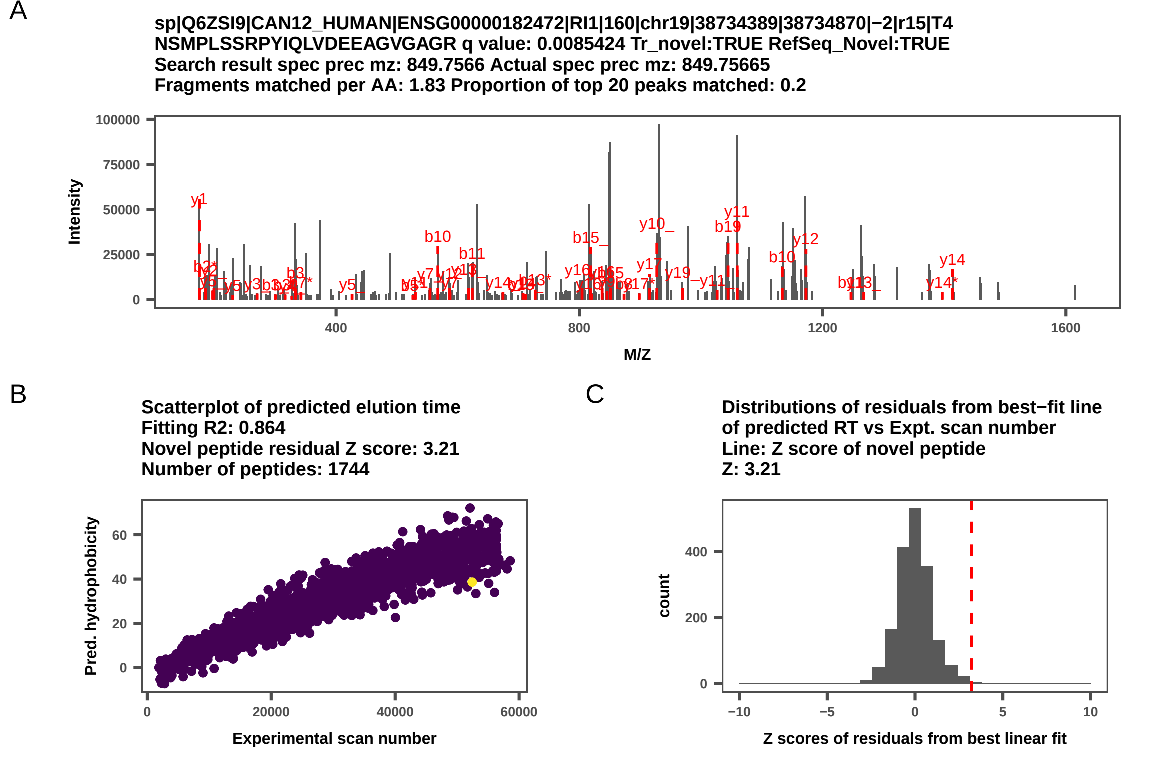
A
sp|Q6ZSI9|CAN12_HUMAN|ENSG00000182472|RI1|160|chr19|38734389|38734870|−2|r15|T4
NSMPLSSRPYIQLVDEEAGVGAGR q value: 0.0085424 Tr_novel:TRUE RefSeq_Novel:TRUE
Search result spec prec mz: 849.7566 Actual spec prec mz: 849.75665
Fragments matched per AA: 1.83 Proportion of top 20 peaks matched: 0.2
Intensity
100000
75000
50000
25000
0
400
800
1200
1600
M/Z
y1
b10
b11
b15_
y10_
y11
b19
y12
b10
y14
y19
y14*
B
Scatterplot of predicted elution time
Fitting R2: 0.864
Novel peptide residual Z score: 3.21
Number of peptides: 1744
Pred. hydrophobicity
60
40
20
0
0
20000
40000
60000
Experimental scan number
C
Distributions of residuals from best−fit line
of predicted RT vs Expt. scan number
Line: Z score of novel peptide
Z: 3.21
count
400
200
0
−10
−5
0
5
10
Z scores of residuals from best linear fit
